# Selection of surgical treatment approaches for cervicothoracic spinal tuberculosis: A 10-year case review

**DOI:** 10.1371/journal.pone.0192581

**Published:** 2018-02-08

**Authors:** Ziqi Zhu, Dingjun Hao, Biao Wang, Wenjie Gao, Ruize Yang, Hua Guo, Yongyi Wang, Lingbo Kong

**Affiliations:** 1 Xi’an Medical University, Beilin District, Xi’an, Shaanxi, China; 2 Department of Spine Surgery, Honghui Hospital Xi’an Jiaotong University College of Medicine, Xi’an, Shaanxi, China; PLOS, UNITED KINGDOM

## Abstract

**Background:**

Cervicothoracic spinal tuberculosis is a rare disease. Due to its difficult and challenging surgical exposure, its surgical treatment approach remains inconclusive. Long-term follow-up studies to address this puzzling issue are rarely seen in the literature. The purpose of this study was to explore the selection of surgical treatment approaches for cervicothoracic spinal tuberculosis through a 10-year case review.

**Methods:**

From January 2003 to January 2013, 45 patients suffering from cervicothoracic spinal tuberculosis were treated surgically. According to the relation between the tuberculosis lesion segments and the suprasternal notch on sagittal MRI, 19 patients were treated with a single-stage anterior debridement, fusion and instrumentation approach, and the other 26 patients were treated with a single-stage anterior debridement and fusion, posterior fusion and instrumentation approach. The clinical efficacy was evaluated using statistical analysis based on the Cobb angle of kyphosis, the Neck Disability Index (NDI) and the Japanese Orthopedic Association (JOA) scoring system. The neurofunctional recovery was assessed by the American Spinal Injury Association (ASIA) system.

**Results:**

All patients were followed up for 6.6 years on average (range 3–13 years). No instrumentation loosening, migration or breakage was observed during the follow-up. The kyphosis angle and NDI and JOA scores were significantly changed from preoperative values of 34.7±6.8°, 39.6±4.6 and 10.7±2.8 to postoperative values of 10.2±2.4°, 11.4±3.6 and 17.6±2.4, respectively (p<0.05). Aside from one recurrent patient, bone fusion was achieved in the other 44 patients within 6 to 9 months (mean 7.2 months). No severe postoperative complications occurred, and patients’ neurologic function was improved in various degrees.

**Conclusions:**

In the surgical treatment of cervicothoracic spinal tuberculosis, single-stage cervical anterior approach with or without partial manubriotomy is capable of complete debridement for tuberculosis lesions. The manner of fixation should be selected based on the anatomical relation of the suprasternal notch and the diseased segments as revealed on sagittal MRI images.

## Introduction

Cervicothoracic spinal tuberculosis is a disease that involves the C7 to T3 levels of the spine, constituting only 5% of all spinal tuberculosis [1–3]. Anatomically, the cervicothoracic junction is a transitional zone between the lordotic cervical spine and the kyphotic thoracic spine. Because the cervicothoracic vertebrae are weight-bearing structures, destruction of these vertebrae by tuberculosis frequently results in spinal instability, severe kyphosis deformity, large paravertebral abscesses and progressive neurological deficit, which cause severe suffering for the patients [4, 5]. Due to the deep location of the cervicothoracic vertebrae, with the sternum, clavicles and mediastinum in the front, the scapulae in the back, and the complicated anatomical relationships of the region, exposure is usually less than satisfactory, making surgical procedures even more challenging [6]. To tackle this issue, a variety of surgical approaches have been proposed. However, it remains inconclusive which surgical treatment approach is optimal for this type of difficult spinal disorder, and long-term follow-up studies to discuss this puzzling issue are rarely seen in the literature. The present study retrospectively reviewed 45 cervicothoracic spinal tuberculosis cases that were surgically treated between January 2003 and January 2013 in our hospital to explore the selection of surgical approaches and clinical outcomes for cervicothoracic spinal tuberculosis patients.

## Materials and methods

### General information

45 patients with cervicothoracic spinal tuberculosis were treated surgically between January 2003 and January 2013, among which 29 were males and 16 were females. The average age was 35.4 (range 17 to 62) years. All patients suffered from neck pain, stiffness and limitation of motion, with low-grade fever, night sweat and weight loss. The average erythrocyte sedimentation rate (ESR) was 67.8 (range 28 to 115) mm/h. C-reactive protein (CRP) was 18.7 (range 10.6 to 55) mmol/l. Eleven of the patients had a prior history of tuberculosis infection. Preoperative imaging showed bone destruction and narrowing of intervertebral spaces of the diseased segments in all patients. In 41 cases, herniation of caseating materials into the spinal canal resulted in spinal canal stenosis and spinal cord compression. Paravertebral cold abscess and retropharyngeal abscess were seen in 37 and 4 cases, respectively. The presurgical cervicothoracic Cobb angle was 34.7±6.8°. The pathologic change regions were as follows: C7/T1 in 8 cases, T1 in 11 cases, T1/T2 in 7 cases, T2 in 6 cases, T2/T3 in 8 cases and T3 in 5 cases. The presurgical neurological and functional classifications were Class A for 2 case, B for 5 cases, C for 9 cases, D for 22 cases and E for 7 cases according to the American Spinal Injury Association (ASIA) system (Table 1).

**Table 1:** Demographic and clinical characteristics of the patients.

Characteristics	Value
Number of patients	45
Age (yrs.)	35.4 (range 17–62)
Male to female ratio	29: 16
History of tuberculosis infection	11
Paravertebral cold abscess	37
Retropharyngeal abscess	4
Erythrocyte sedimentation rate (mm/h)	67.8 (range 28–115)
C-reactive protein (mmol/l)	18.7 (range 10.6–55)
Associated with neurological damage	38
Patient classification (segments)	
Group A	19 (C7/T1 in 8, T1 in 11)
Group B	13 (T1/T2 in 7, T2 in 6)
Group C	13 (T2/T3 in 8, T3 in 5)

### Presurgical preparation

Isoniazid, rifampicin, pyrazinamide and streptomycin were administered to each patient for a course of over 2 weeks before surgery. Supportive therapies were also given to address anemia and hypoproteinemia. Surgeries were performed when ESR either significantly decreased or fell below 60 mm/h.

### Patient classification

Patient classification in the present study was based on the location of the tuberculosis lesion in relation to the suprasternal notch on sagittal Magnetic Resonance Imaging (MRI) images, as follows:

A The tuberculosis focus was located higher than the suprasternal notch level.
B The tuberculosis focus lay exactly on the suprasternal notch level.
C The tuberculosis focus was located lower than the suprasternal notch level.

**Fig 1:**
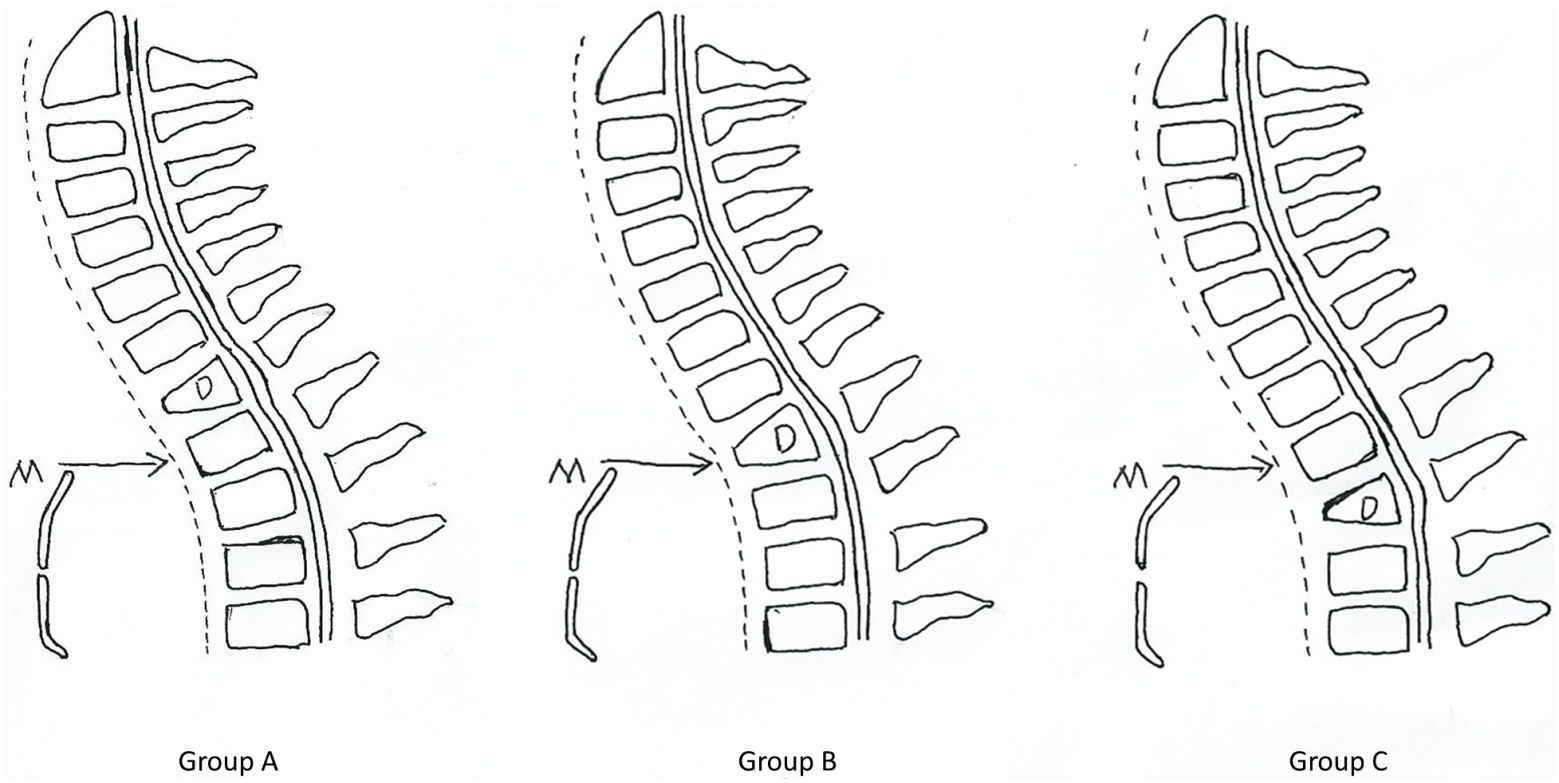
Classification of patients. Group A: the tuberculosis focus was located higher than the suprasternal notch level. Group B: the tuberculosis focus lay exactly on the suprasternal notch level. Group C: the tuberculosis focus was located lower than the suprasternal notch level. D: diseased segments. M: manubrium. Arrow: the suprasternal notch level.

19, 13 and 13 cases fell into Groups A, B and C in the present study, respectively (Fig 1).

### Surgical approaches

**Fig 2:**
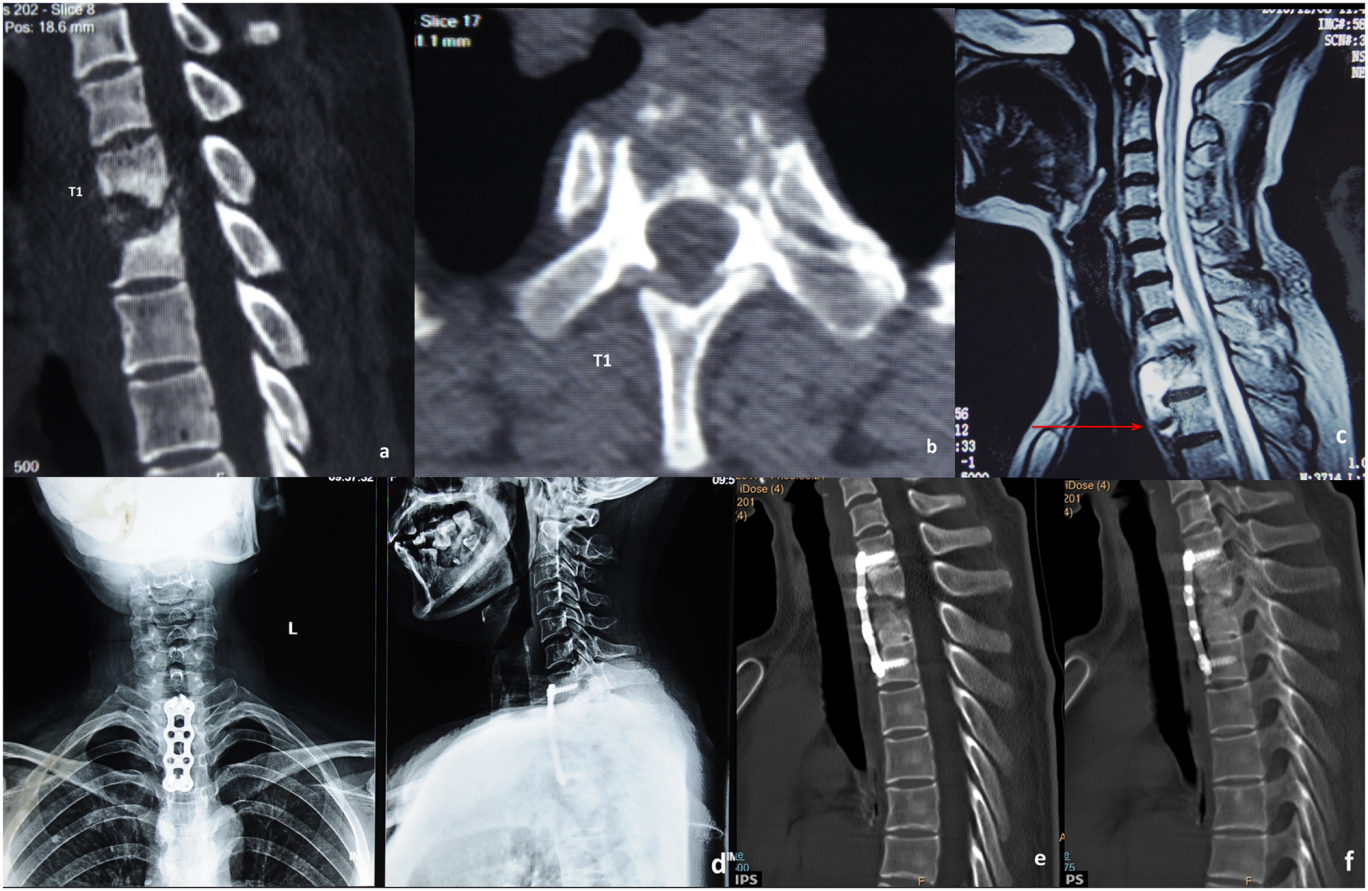
A typical case for group A. A 56-year-old patient’s preoperative CT scanning shows destructive segments located at the T1 segments with corrasion of the T1 vertebra (a-b). Preoperative sagittal MRI shows that the tuberculosis focus is located higher than the suprasternal notch level (c). One-week postoperative X-ray image shows internal fixation in good position (d). Six-month postoperative CT scanning reveals no cervicothoracic anterior graft fusion yet (e). Three-year postoperative CT scanning reveals cervicothoracic anterior graft fusion (f).

**Fig 3:**
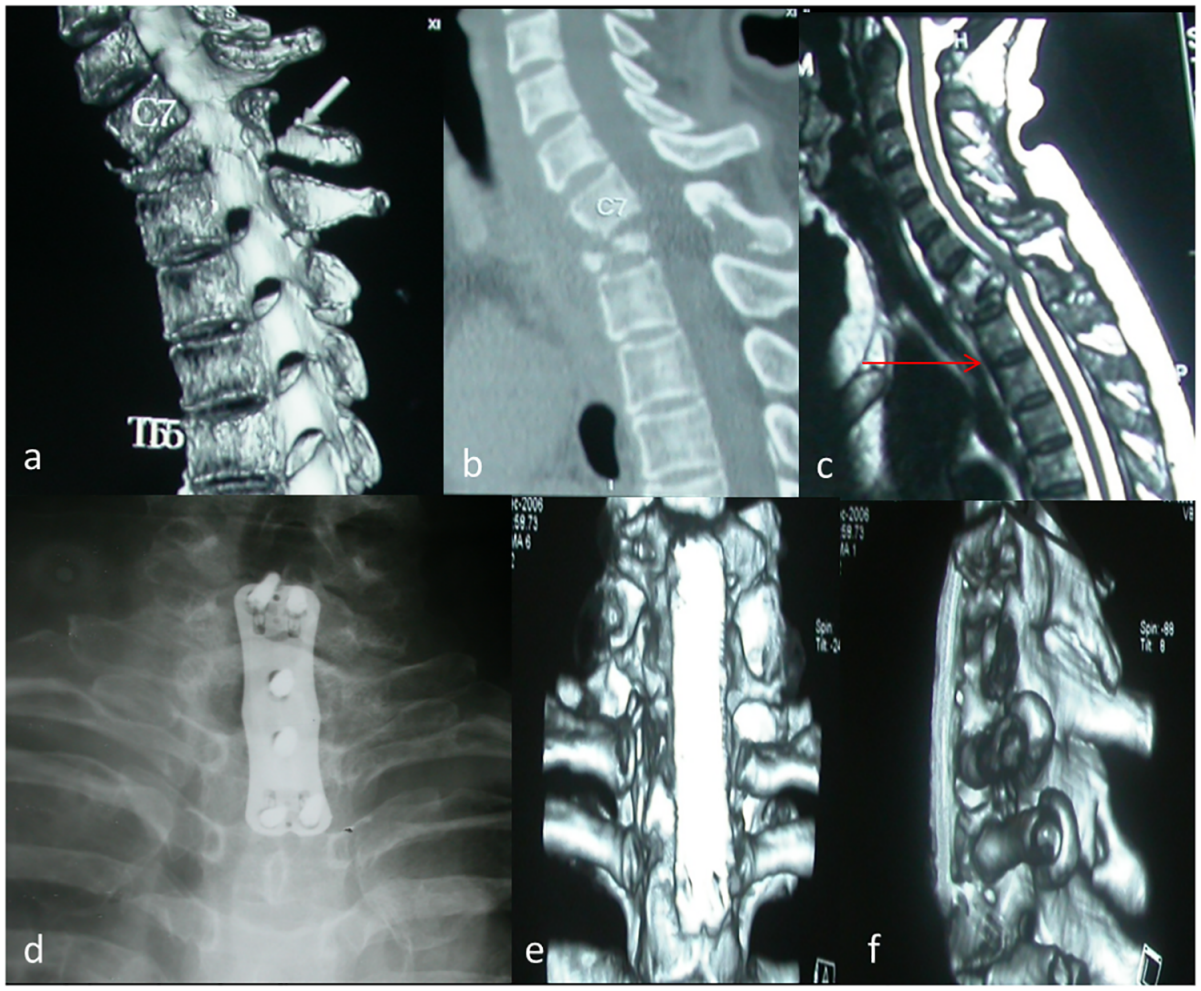
Another typical case for group A. A 45-years-old patient’s preoperative CT scanning shows destructive segments located at C7/T1 segments with collapse of T1 vertebra (a-b). Preoperative sagittal MRI shows the tuberculosis focus is located higher than the suprasternal notch level (c). One-week postoperative X-ray image shows internal fixation in good position (d). Three years postoperative CT scanning reveals cervicothoracic anterior graft fusion (e-f).

Patients in group A were treated with a single-stage anterior debridement and intervertebral fusion with instrumentation. Fusion was performed at the levels above, below, and through the diseased vertebral bodies. The patient was placed supine on the operation table, and general endotracheal anesthesia was induced. Selection of a right-sided or left-sided approach was based on the location of paravertebral abscess. In the case that both sides had equivalent amounts of abscess, a left-sided approach was preferred to avoid the possibility of injuring the recurrent laryngeal nerve. An oblique incision was made along the anterior border of the sternocleidomastoid (SCM) to the sternal notch. The omohyoid was transected, and the SCM was retracted outwards to expose the prevertebral fascia through the interval between the carotid sheath and the trachea and esophagus. Structures posterior to the sternum were dissected with a finger following its exposure. Dissection was then carried down between the carotid sheath and the esophagus and trachea. A retractor was employed to divide the brachiocephalic trunk and left common carotid artery to expose the prevertebral fascia of the cervicothoracic spine. The fascia and abscess wall were carefully incised, and the pus was suctioned thoroughly. The caseous necrotic tissue, dead bone and damaged intervertebral disc were thoroughly removed. Intraspinal herniated tissue was also removed to relieve spinal cord compression completely. A curette was used to thin the graft bed until leakage of blood was observed. Under monitoring of somatosensory evoked potentials (SSEPs), intervertebral retraction was performed to correct kyphosis. An appropriately sized autogenous bone graft harvested from the iliac crest was placed into the graft bed. Streptomycin powder was applied locally, followed by anterior instrumentation of a titanium plate. A typical case is described in Figs 2 and 3.

**Fig 4:**
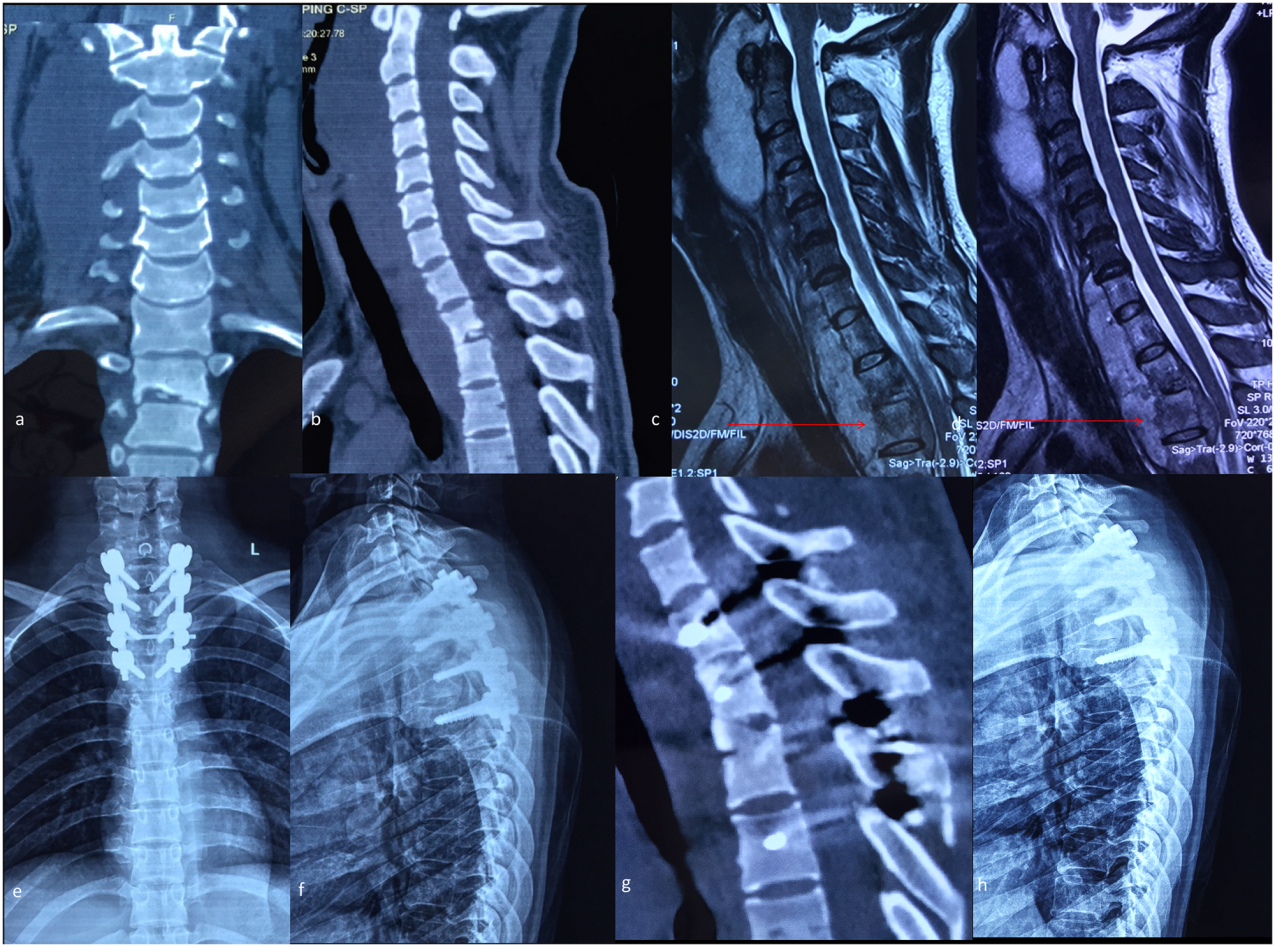
A typical case for group B and C. A 27-year-old patient’s preoperative CT scanning shows destructive segments located at the T2/3 segments (a-b). Preoperative MRI shows a huge paravertebral abscess located in front of the vertebral bodies and the compression of the spinal cord, while the tuberculosis focus lies exactly on the suprasternal notch level (c-d). Two-week postoperative antero-posterior and lateral plain radiograph shows the internal instruments in a satisfactory position (e-f). Four-year postoperative CT scanning demonstrates that the cervicothoracic fusion is consolidated completely (g). Six-year postoperative lateral plain radiograph shows no instrumentation loosening, migration or breakage (h).

**Fig 5:**
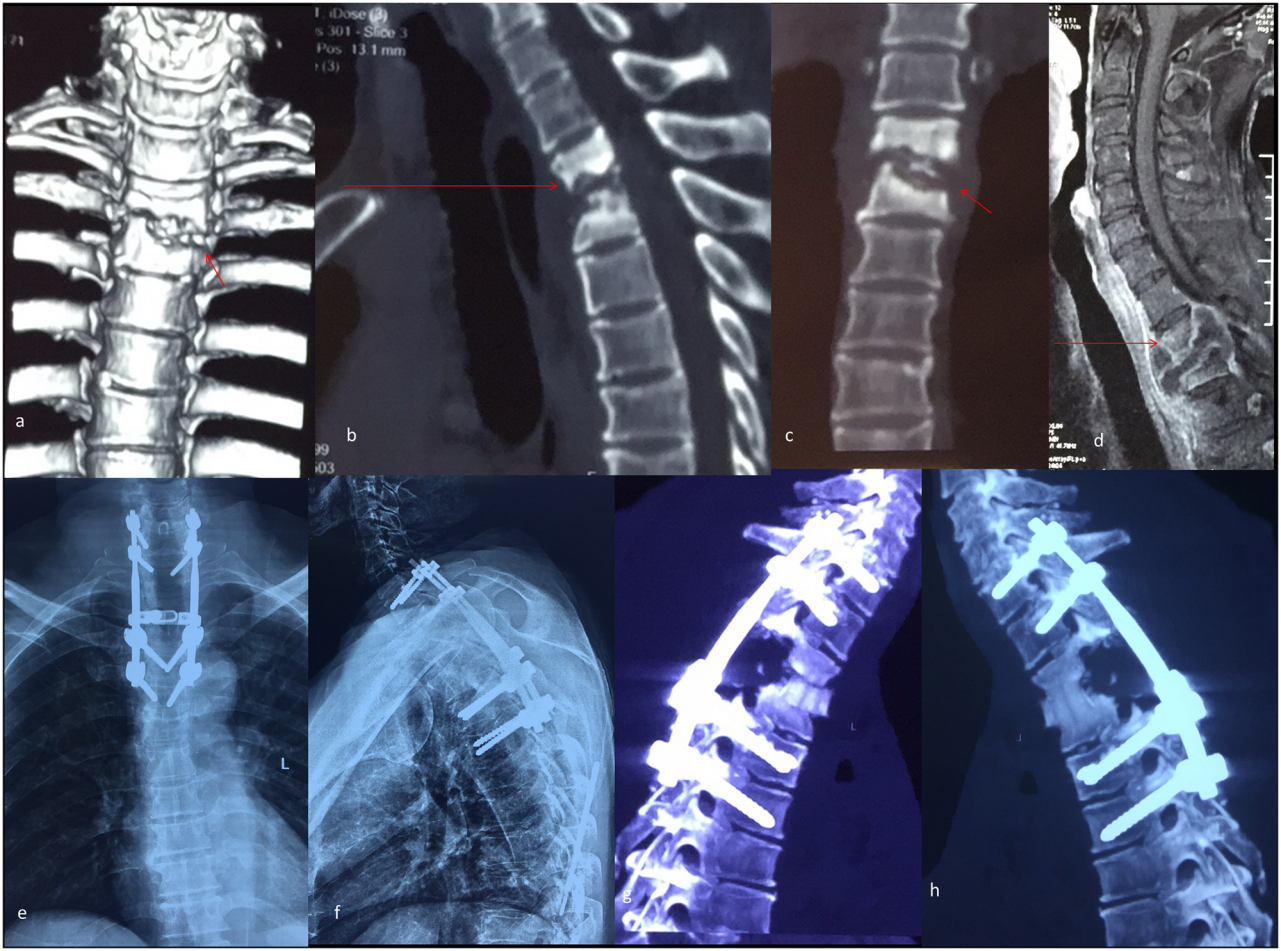
Another typical case for group B and C. A 38-year-old patient’s preoperative CT scanning shows destructive segments located at the T2/3 segments (a-c). Preoperative MRI shows the tuberculosis focus lies lower than the suprasternal notch level (d). Two-week postoperative antero-posterior and lateral plain radiograph shows the internal instruments in a satisfactory position (e-f). Six-month postoperative CT scanning shows the anterior bone grafting is in a good position but without fusion (g). Five-year postoperative CT scanning demonstrates that the cervicothoracic fusion is consolidated completely (h).

For patients in groups B and C, anterior debridement and fusion with posterior fusion and instrumentation was performed. The anterior procedure was identical to that of group A patients, except that in 6 patients median manubriotomy was adopted for a more favorable exposure to make distal tuberculosis lesions accessible. In posterior procedures, fixation was performed at one level above and below the diseased vertebrae. Pedicle screws were applied, while interlaminar grafting and bilateral facet joint grafting were performed to obtain optimal stability. A typical case is described in Figs 4 and 5.

### Postsurgical treatments

After surgery, the patients were routinely administered antibiotics for 48 hours as well as corticosteroids and dehydrants for 72 hours. Patients were discharged when their conditions were stable and were asked to wear a cervicothoracic brace for 3 months afterwards. Isoniazid, rifampin, pyrazinamide and streptomycin quadruple chemotherapy was administered for 3 months, after which streptomycin was replaced with ethambutol and the therapy was continued for another 9 to 15 months. ESR, CRP, hepatic and renal function tests were ordered upon monthly visits.

### Therapeutic effect assessment

X-ray and computed tomography (CT) scans were performed at 3, 6, 9, 12, 18, and 24 months and every 6 months thereafter to evaluate the position of the internal fixation, presence of loosening, migration, graft bone fusion, recurrence of tuberculosis and change in the cervicothoracic Cobb angle. Symptomatic and neurofunctional recovery after surgery was evaluated according to the NDI and JOA scoring systems as well as the ASIA system.

### Statistical analysis

We adopted Student’s t-test to examine the NDI scores, JOA scores and Cobb angles before surgery and at the final follow-up after surgery. To avoid bias in collecting Cobb angles and scores caused by multiplayer, data were collected by one author who is an attending physician and specializes in the diagnosis and treatment of spinal disorders. The Statistical Package for the Social Sciences version 16.0 (SPSS, Chicago, IL, USA) was used for the statistical analysis. p<0.05 denotes a statistically significant difference, and the descriptive data were presented as the mean ± standard deviation.

### Statement

This study was approved by the Xi’an Honghui Hospital Ethics Committee, and all 45 patients provided written informed consent of participating in the study. The consent for figures publication included in this paper has been obtained from the corresponding patients.

## Results

The mean surgical duration was 178 minutes (range 65 to 270 minutes). Intraoperative blood loss was on average 590 milliliters (range 100 to 1200 milliliters). No injury of the major vessels or the spinal cord was observed. Esophageal laceration occurred in 1 patient as a result of excessive retraction during surgery; the damage was repaired immediately and did not result in esophageal fistula or dysphagia. One patient presented with dysphonia and bucking after surgery, laryngoscopy revealed glottal insufficiency, indicating an injury of the recurrent laryngeal nerve and superior laryngeal nerve. However, at the 3-month follow-up, the symptoms were relieved. No operation-associated complications were observed in any other patients. The mean follow-up period was 6.6 years (range 3 to 13 years). One patient in group A who received single-stage anterior debridement, intervertebral fusion and internal fixation presented with a sinus of the incision, representing recurrent tuberculosis at the 3-month follow-up. This particular patient then received one-stage anterior instrumentation removal, radical debridement and intervertebral fusion, second-stage posterior fusion and instrumentation. Fusion of the cervicothoracic segment was achieved at 6-month follow-up, and after a follow-up period of more than 5 years, there was no recurrent tuberculosis. Bony fusion was achieved in the other 44 patients within 6 to 9 months (mean 7.2 months) postoperatively. During the follow-up, no instrumentation loosening, migration or breakage was observed. The cervicothoracic Cobb angle was significantly decreased to 10.2±2.4° at final follow-up, and the difference between the preoperative and postoperative Cobb angles was statistically significant (P<0.05). The mean NDI score and JOA score were changed from 39.6±4.6 and 10.7±2.8 preoperatively to 11.4±3.6 and 17.6±2.4 at final follow-up, respectively. The differences between preoperative and postoperative NDI and JOA scores were both statistically significant (P<0.05). The detailed statistical results are shown in Table 2. Aside from the patient with recurrent tuberculosis, the remaining 44 patients’ ESR and CRP returned to normal within three months’ follow-up. In 38 cases complicated with neurological impairment, 29 (76%) showed various degrees of neurological functional recovery; detailed ASIA scores are shown in Table 3.

**Table 2:** Statistical results in the kyphosis Cobb angle, NDI score and JOA score.

	Preoperative	Postoperative	p
Kyphosis Cobb angle (°)	34.7±6.8	10.2±2.4	<0.05
NDI score	39.6±4.6	11.4±3.6	<0.05
JOA score	10.7±2.8	17.6±2.4	<0.05

NDI score: Neck Disability Index score. JOA score: Japanese Orthopaedic Association score.

Data are presented as mean±standard deviation.

**Table 3:** Neurological functional recovery according to the ASIA system.

Preoperative	Postoperative				
	A	B	C	D	E
A	1			1	
B		2	1	2	
C			3	3	3
D				3	19
E					7

## Discussion

Exposure of the cervicothoracic junction continues to be a challenge in spinal surgeries. Compared to the anterior approach, the posterior approach may appear to be simpler, but it makes no sense because the majority of lesions are situated in the anterior vertebral bodies. Consequently, the anterior approach is widely accepted. With bony structures including the sternum, clavicles and ribs in the front and large vessels, the thoracic duct and important nerves nearby, it is difficult to expose the cervicothoracic junction anteriorly, and the procedure is accompanied with high risks. Meanwhile, patient suffering from cervicothoracic tuberculosis is usually complicated with regional kyphosis caused by bony structure destruction, which presents a further challenge to the surgeons [7–11]. As a result, for patients with cervicothoracic tuberculosis, it is essential to individualize specific surgical approaches based on the location of lesions to obtain optimal exposure and maximize safety for the patients.

Currently, approaches to the cervicothoracic spine include conventional anterior cervical approach, anterior approach combined with sternotomy or manubriotomy, standard transclavicular approach, approach by resection of the manubrio-clavicular complex, anterolateral transthoracic approach, and combined approaches. The conventional anterior cervical approach is the simplest one, inflicting minimal damage to adjacent structures, and thus yields early patient recovery. The procedure is capable of exposing down to the T1-T2 segments. However, in regard to the T3 segment diseases or surgeries that involve stabilization down to the T3 segment, the approach may not provide sufficient exposure. Other approaches may obtain optimal T3 segment exposure, but the procedures themselves are complicated and patients suffer from high morbidity and high risk of mediastinal infections, bone defects and nonunion, which to a great extent impair the therapeutic effect of the surgery [12, 13].

Sharan et al [14] revealed that the T1-T2 disc could be visualized in its entirety above the suprasternal notch in 45.28% of patients but that the T2-T3 disc could be exposed only in 14.15% from a radiographic analysis. To expose the T3 vertebra, anterior approach with manubriotomy would be necessary in 80% to 85% cases [14–17]. Kaya RA et al [2] reported the successful utilization of conventional anterior cervical approach in T2 vertebral instrumentation and T3 vertebral decompression in long-neck patients. They proposed that in a selected group of patients, conventional anterior cervical approach achieved sufficient exposure for debridement of cervicothoracic segments. In the present study, anterior approaches were performed on all 45 patients for debridement; 19 patients had anterior titanium plate fixation, and the other 26 patients had posterior pedicle screw fixation. Our experience showed that for patients with cervicothoracic spinal tuberculosis, presurgical MRI scanning should be a routine exam to determine the anatomical relation between the suprasternal notch and the diseased spinal segments. For patients with lesions higher than suprasternal notch or planned for internal fixation at the suprasternal notch level, single-stage anterior debridement, bone grafting, fusion and instrumentation yielded satisfactory therapeutic effect. For lesions lower than the suprasternal notch level or planned for internal fixation below the suprasternal notch level, single-stage anterior debridement and fusion, posterior fusion and instrumentation approach would be a safe and effective option.

To debride tuberculosis foci located below the suprasternal notch level, an extensive prevertebral fascia dissection was preferred to achieve complete debridement. In short-neck patients or those with a long sternum, debridement might be difficult. In this circumstance, a partial median manubriotomy is preferred to obtain a larger surgical field and achieve complete debridement of remote lesions. In the present study, 6 patients had a short neck; thus, a conventional anterior cervical approach was insufficient for clearance of remote lesions in the T3 segment. We thus performed a partial median manubriotomy to achieve complete debridement.

For lesions involving the T3 vertebral body, it is possible to achieve debridement through conventional anterior cervical approach with or without partial manubriotomy, but internal fixation is impractical due to the blockade of nearby bony structures. In such cases, anterior approach for internal fixation requires full sternotomy, which demands high technical proficiency, leading to increased morbidity and extending the recovery time of the patient after surgery. However, approaching by resection of the manubrio-clavicular complex might induce shoulder girdle dysfunction [7, 17–20]. Given the above considerations, we performed anterior debridement, bone grafting and fusion, posterior bone grafting and internal fixation on these patients. In the present study, 8 patients had lesions at the T2/3 segments, and 5 patients had lesions at the T3 segment, which were below the suprasternal notch levels. We performed one-stage combined anterior and posterior approaches as described above. After a follow-up period of over 3 years, all 13 patients presented with anteroposterior 360° fusion of the spine and none had recurrent spinal tuberculosis. Our findings demonstrated that anterior cervical approach with partial manubriotomy was capable of exposing the T3 level and allowing for a complete debridement. Without the need for full sternotomy or clavicular resection or entering the pleural cavity, the procedure not only has a lower impact on respiration and circulation but also takes less time and is less likely to induce morbidity, benefiting the patients’ recovery after surgery.

The anterior approaches are capable of restoring the stability and physiological curvatures of the spine through complete debridement and autogenous bone grafting, and they prevent the loss of physiological curvature post-surgically. In the present study, we performed an intervertebral retraction after debridement to correct kyphosis of the cervicothoracic spine induced by tuberculosis. Anterior approaches also allowed for complete clearance of abscesses and dead bone in the spinal canal. Such direct decompression functioned positively in the patients’ neurofunctional recovery after surgery. Of the 38 patients who had presented with neurological impairment, 29 showed various degrees of neurofunctional improvement after surgery.

## Conclusion

To summarize, in the surgical treatment of cervicothoracic spinal tuberculosis, single-stage cervical anterior approach with or without partial manubriotomy is capable of complete debridement for tuberculosis lesions. Meanwhile, it is relatively simple and induces less morbidity. The manner of fixation should be selected based on the anatomical relation of the suprasternal notch and the diseased segments as revealed on sagittal MRI images.

## Supporting information

S1 File Editorial certificate. The editorial certificate of American Journal Experts.
(PDF)

S2 File Consent form. A blank copy of the consent form used.
(DOCX)
