# Supplementary material for: Selection of surgical treatment approaches for cervicothoracic spinal tuberculosis: A 10-year case review
Source: PLoS One. 2018 Feb 8;13(2):e0192581. doi: 10.1371/journal.pone.0192581 (PMC5805302; doi:10.1371/journal.pone.0192581)
Supplement: S2 File — A blank copy of the consent form used. (DOCX) [file pone.0192581.s002.docx]

知情同意书

尊敬的入选患者：

您的医学资料已入选“颈胸段脊柱结核手术治疗策略——10年病例回顾”（Selection of Surgical Treatment Approaches for Cervicothoracic Spinal Tuberculosis: 10-years cases reviewed）研究项目。望您同意参与本项科学研究并可能发表您的影像资料。

您的意见：

西安交通大学附属红会医院

2016年1月20日
